# Supplementary material for: Causal Relationship between Aging and Anorexia Nervosa: A White-Matter-Microstructure-Mediated Mendelian Randomization Analysis
Source: Biomedicines. 2024 Aug 16;12(8):1874. doi: 10.3390/biomedicines12081874 (PMC11351342; doi:10.3390/biomedicines12081874)
Supplement: Supplementary file 1 [file biomedicines-12-01874-s001.zip › Supplementary Figure/Figure S4/Singe SNP plot of MR analysis for TL to ubm-b-1752.pdf]

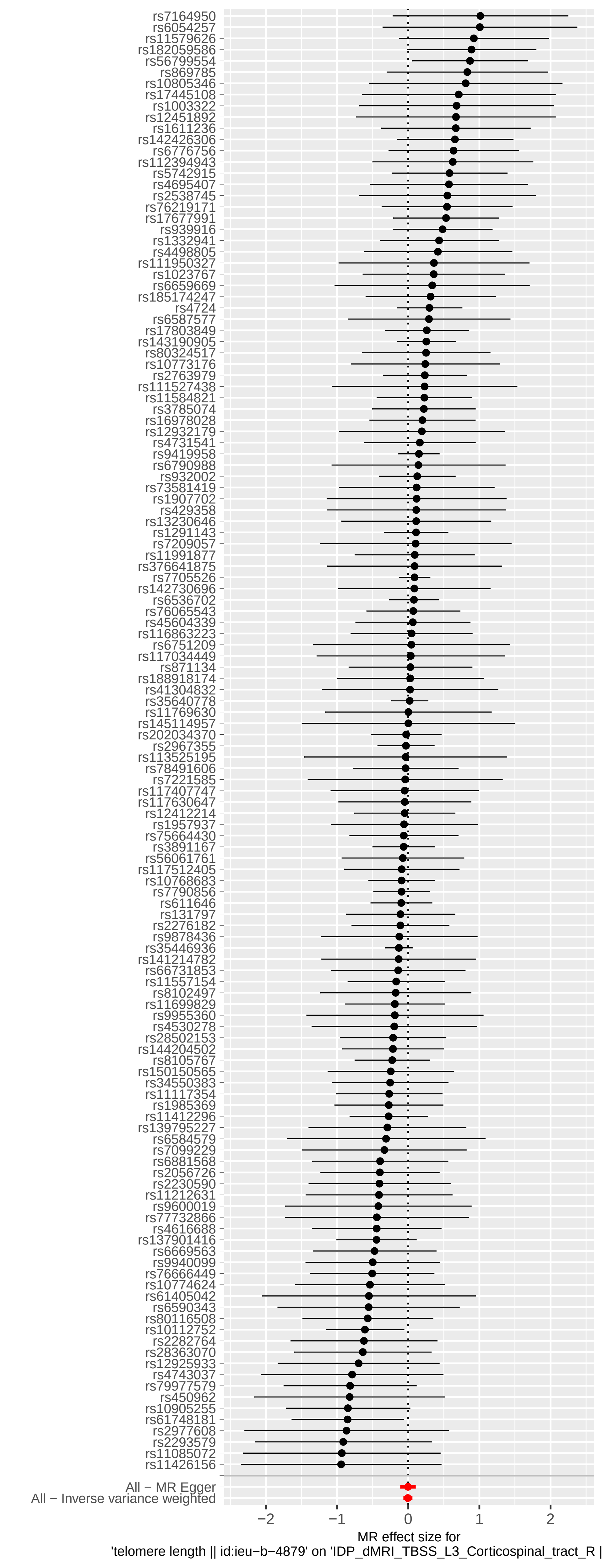
rs7164950
rs6054257
rs11579626
rs182059586
rs56799554
rs869785
rs10805346
rs17445108
rs1003322
rs12451892
rs1611236
rs142426306
rs6776756
rs112394943
rs5742915
rs4695407
rs2538745
rs76219171
rs17677991
rs939916
rs1332941
rs4498805
rs111950327
rs1023767
rs6659669
rs185174247
rs4724
rs6587577
rs17803849
rs143190905
rs80324517
rs10773176
rs2763979
rs111527438
rs11584821
rs3785074
rs16978028
rs12932179
rs4731541
rs9419958
rs6790988
rs932002
rs73581419
rs1907702
rs429358
rs13230646
rs1291143
rs7209057
rs11991877
rs376641875
rs7705526
rs142730696
rs6536702
rs76065543
rs45604339
rs116863223
rs6751209
rs117034449
rs871134
rs188918174
rs41304832
rs35640778
rs11769630
rs145114957
rs202034370
rs2967355
rs113525195
rs78491606
rs7221585
rs117407747
rs117630647
rs12412214
rs1957937
rs75664430
rs3891167
rs56061761
rs117512405
rs10768683
rs7790856
rs611646
rs131797
rs2276182
rs9878436
rs35446936
rs141214782
rs66731853
rs11557154
rs8102497
rs11699829
rs9955360
rs4530278
rs28502153
rs144204502
rs8105767
rs150150565
rs34550383
rs11117354
rs1985369
rs11412296
rs139795227
rs6584579
rs7099229
rs6881568
rs2056726
rs2230590
rs11212631
rs9600019
rs77732866
rs4616688
rs137901416
rs6669563
rs9940099
rs76666449
rs10774624
rs61405042
rs6590343
rs80116508
rs10112752
rs2282764
rs28363070
rs12925933
rs4743037
rs79977579
rs450962
rs10905255
rs61748181
rs2977608
rs2293579
rs11085072
rs11426156
All − MR Egger
All − Inverse variance weighted
−2
−1
0
1
2
MR effect size for
'telomere length || id:ieu−b−4879' on 'IDP_dMRI_TBSS_L3_Corticospinal_tract_R |
